# Clozapine-induced stuttering in the absence of known risk factors: a case report

**DOI:** 10.1186/s13256-021-02803-8

**Published:** 2021-04-17

**Authors:** Florence Jaguga

**Affiliations:** Moi Teaching & Referral Hospital, P.O. BOX 3-30100, Eldoret, Kenya

**Keywords:** Clozapine, Stuttering, Risk factor, Case report

## Abstract

**Background:**

Stuttering is a rare side effect of clozapine. It has been shown to occur in the presence of one or more factors such as abnormal electrophysiological findings and seizures, extrapyramidal symptoms, brain pathology, and a family history of stuttering. Few case reports have documented the occurrence of clozapine-induced stuttering in the absence of these risk factors.

**Case presentation:**

A 29-year-old African male on clozapine for treatment-resistant schizophrenia presented with stuttering at a dosage of 400 mg/day that resolved with dose reduction. Electroencephalogram findings were normal, and there was no clinical evidence of seizures. The patient had no prior history or family history of stuttering, had a normal neurological examination, and showed no signs of extrapyramidal symptoms.

**Conclusion:**

Clinicians ought to be aware of stuttering as a side effect of clozapine, even in the absence of known risk factors. Further research should investigate the pathophysiology of clozapine-induced stuttering.

## Background

Clozapine is an atypical antipsychotic with proven efficacy in the management of treatment-resistant schizophrenia [1]. It has well-documented side effects that include agranulocytosis, myocarditis, seizures, metabolic side effects, and hypersalivation [1]. Stuttering, a disorder that affects fluency of speech, is a rare side effect of clozapine that has been reported in a number of case studies [2–9]. The pathophysiologic mechanisms underlying stuttering remain unresolved but have been postulated to include dopamine dysregulation, genetic mechanisms, and structural and functional brain changes [10]. Not surprisingly, prior case reports have found clozapine-induced stuttering to co-occur with extrapyramidal symptoms [4, 5], seizure activity [5, 6, 11, 12], brain pathology [4, 5, 13], and a family history of stuttering [4]. Few case reports have documented clozapine-induced stuttering in the absence of these risk factors [3].

The aim of this case report is to describe a case of clozapine-induced stuttering that occurred in the absence of seizure activity, extrapyramidal symptoms, and family history of stuttering.

## Case presentation

The patient, a 29-year-old African male, was admitted in September 2019 and diagnosed with schizophrenia and comorbid cannabis and tobacco use disorders. At the time of admission, he reported a 7-year history of auditory hallucinations that were derogatory in nature, paranoid thoughts, and a lack of motivation. In addition, he reported experiencing his thoughts as though they were someone else’s and had beliefs that his thoughts were being taken out of his mind. During the 7-year period, he had been on varying doses of oral olanzapine (2.5–10 mg/day) and fluphenazine long-acting injectable (25–37.5 mg monthly) on and off, with the two being given in combination at certain times. He reported to have been using an average of one roll of cannabis and five cigarettes per day to “feel better.”

He was initially admitted into a psychiatric ward for stabilization of acute psychotic symptoms. This was immediately followed by admission into an in-patient substance use disorder rehabilitation unit for tobacco and cannabis use disorders. Altogether, he was hospitalized for a continuous period of 5 months up to February 2020.

During this time, with supervised medication administration and no substance use, the patient failed adequate trials of oral olanzapine at 20 mg/day and oral quetiapine at 800 mg/day (about 9 weeks each). He reported persistent auditory hallucinations, delusions of reference, delusions of thought possession, and a lack of motivation. A brief trial of oral risperidone at 6 mg/day was attempted for 2 weeks but was halted by a worsening of the psychotic symptoms. Consequently, a decision to initiate clozapine was made. During the last 2 weeks of hospitalization, oral clozapine was started at a dosage of 25 mg/day. This was escalated by 25 mg/day up to a dosage of 400 mg/day in divided doses by the end of the second week. We utilized the United States Food and Drug Administration (FDA)-approved prescribing guidelines for clozapine administration and titration [14] in this patient. At 400 mg/day of clozapine, the patient reported no psychotic symptoms, had improvements in motivation, and was discharged from the hospital. In addition to the antipsychotics, the patient was on nicotine gum at 8–12 mg/day and bupropion at 150 mg/day throughout the period of hospitalization. The bupropion and nicotine gum were, however, dropped at discharge to allow for finances to be spent on the purchase of clozapine. He had no cravings for tobacco and cannabis at the time of discharge.

Two weeks after discharge from hospital, while on clozapine only, the patient presented with a dysfluency characterized by random hesitations during speech and repetitions of speech sounds. This caused him to avoid social interaction. The patient had no prior history or family history of stuttering. Clinically, he had no extrapyramidal symptoms and no evidence of seizures. A neurological examination and the findings of an electroencephalogram (EEG) were both normal. The patient reported no tobacco or cannabis use after discharge from hospital, and this was corroborated by family. We did not perform a urine drug screen since the procedure was costly and not affordable to the patient.

The author was not aware of stuttering as a side effect of clozapine at the time. A literature search was done that identified a number of case reports on the subject. This prompted the clinician to consider a diagnosis of clozapine-induced stuttering. A gradual dose reduction of clozapine was attempted based on reports indicating this as an effective strategy for clozapine-induced stuttering [2, 6]. Two weeks after onset of dose reduction, while at 200 mg/day, the stuttering stopped but the psychotic symptoms reemerged. A second trial of a dose escalation, but more gradual this time (12.5 mg on alternate days), was halted 2 weeks later by reemergence of stuttering and persistence of psychotic symptoms at 300 mg/day. This prompted the use of combination therapy with oral aripiprazole (10 mg/day) and oral clozapine (200 mg/day). Such a combination has been shown to be safe and effective for patients experiencing suboptimal response with clozapine [15–17]. At the time of writing this paper, the patient has been on the aripiprazole/clozapine combination for 4 weeks. The patient reports a reduction in intensity of psychotic symptoms and a complete resolution of stuttering.

## Discussion and conclusions

This case supplements four case reports that have shown the occurrence of clozapine-induced stuttering in the absence of known risk factors. Murphy *et al.* [3] reported three cases of clozapine-induced stuttering that occurred without any evidence of seizures, movement disorders, or prior history of stuttering [3]. Similarly, Nagendrappa *et al.* [2] reported clozapine-induced stuttering in a 29-year-old male in the absence of a childhood developmental disorder, sensory deficits, focal neurological deficits, and seizure activity.

The exact pathophysiological mechanism(s) underpinning clozapine-induced stuttering remain unknown. Several case reports suggest an association between clozapine-induced stuttering and seizure activity. Rachamallu *et al.* [5] reported epileptiform discharges on the EEG recordings of a 16-year-old male with clozapine-induced stuttering. Similarly, Duggal *et al.* [11] reported abnormal EEG findings and generalized tonic-clonic seizures in a 28-year-old male on clozapine who developed stuttering. Both Begum [12] and Supprian *et al.* [7] reported myoclonic jerks in patients with clozapine-induced stuttering. In all the above cases, the stuttering and seizures resolved with administration of valproate. Our patient had normal EEG findings and no clinical evidence of seizure activity. We therefore did not offer an anticonvulsant.

Stuttering from clozapine has also been postulated to arise from extrapyramidal reactions to the medication. Interestingly, extrapyramidal symptoms have been reported to co-occur with seizures in patients with clozapine-induced stuttering Thomas *et al.* [18], for example, reported dyskinesia and abnormal EEG recordings in a patient who developed stuttering while on clozapine. Similarly, Rachamallu *et al.* [5] reported the co-occurrence of seizures and dyskinesia in a patient with clozapine-induced stuttering. In the latter case, valproate resolved the stuttering, seizures, and dyskinesia, suggesting seizure activity as a primary underlying mechanism for clozapine-induced stuttering [5]. In one case report, a patient on clozapine who developed stuttering and orofacial dyskinesia in the absence of seizure activity failed to respond to tetrabenazine [4]. This further casts doubt on the role of extrapyramidal mechanisms in the etiology of clozapine-induced stuttering. Our patient had no extrapyramidal symptoms throughout the period of clozapine use.

Other less well-documented risk factors for clozapine-induced stuttering include brain pathology and a family history of stuttering. A 16-year-old patient who developed stuttering while on clozapine had a history of brain contusion at the age of 14 years. [5]. Similarly, a 55-year-old male with schizophrenia who developed clozapine-induced stuttering had a history of head injury 14 years prior as well as a family history of stuttering [4]. Both patients, however, had normal brain magnetic resonance imaging (MRI) findings at the time of stuttering onset [4, 5]. A neurological examination yielded no positive findings in our patient.

Psychoactive substance use may affect clozapine metabolism. Specifically, abrupt tobacco and cannabis smoking cessation has been reported to result in increased plasma clozapine levels and to contribute to clozapine toxicity [19]. In our case, the patient denied the use of either tobacco or cannabis after discharge from hospital. Even though this was corroborated by family, urine drug levels could have been a more objective measure of use. We therefore cannot rule out tobacco and cannabis withdrawal as potential contributors to the stuttering.

Dose reduction for treating clozapine-induced stuttering has been reported to be successful at controlling the side effect without psychotic symptom decompensation [2, 6, 9]. In our case, the patient experienced psychotic symptoms at clozapine doses that did not induce stuttering and experienced stuttering at doses sufficient to produce psychotic symptom resolution. Titration of clozapine dosage to achieve a remission of both stuttering and psychotic symptoms was therefore unsuccessful, limiting the use of clozapine at therapeutic doses.

Limitations to this report are that urine drug screen levels for tobacco and cannabis were not checked at the time of stuttering since these were not affordable for the patient. In addition, we did not perform prolonged EEG monitoring and can therefore not completely rule out seizure activity.

This case emphasizes the need for clinicians to be aware of clozapine-induced stuttering, even in the absence of known risk factors. Further, it highlights the need for future research to investigate the pathophysiology of clozapine-induced stuttering.

## Data Availability

Not applicable

## Abbreviations

EEG: Electroencephalogram
FDA: Food and Drug Administration
mg: Milligrams
MRI: Magnetic resonance imaging
